# Construction of a ferroptosis-related eight gene signature for predicting the prognosis and immune infiltration of thyroid cancer

**DOI:** 10.3389/fendo.2022.997873

**Published:** 2022-11-04

**Authors:** Xiaoying Ren, Huijun Du, Weilun Cheng, Yujing Wang, Yuanxin Xu, Shuang Yan, Yunan Gao

**Affiliations:** ^1^ Department of Endocrinology and Metabolism, The Fourth Affiliated Hospital of Harbin Medical University, Harbin, China; ^2^ Department of Cardiology, The Fourth Affiliated Hospital of Harbin Medical University, Harbin, China; ^3^ Department of General Surgery, The Second Affiliated Hospital of Harbin Medical University, Harbin, China

**Keywords:** thyroid cancer, ferroptosis-related genes, prognosis, tumor microenvironment, immune infiltration

## Abstract

**Background:**

Thyroid cancer is the most common malignant tumor of the endocrine system. Most patients with thyroid cancer have a good prognosis, although a small proportion experience recurrence and metastasis and have a poor prognosis. Ferroptosis is a novel form of regulated cell death (RCD); previous studies have confirmed that ferroptosis was associated with thyroid cancer. The purpose of this study was to investigate the key ferroptosis-related genes in thyroid cancer and their relationship with prognosis and immune cell infiltration.

**Methods:**

In this study, 497 thyroid cancer RNA expression datasets were downloaded from the cancer genome atlas (TCGA) cohort and a prognostic risk model for eight ferroptosis-related genes (FRGs) was constructed by Lasso-Cox regression. The prognostic value of the risk model and the correlation of prognostic features with immune scores and tumor immune cell infiltration were systematically analyzed.

**Results:**

The prognostic risk model for eight FRGs (*DPP4*, *TYRO3*, *TIMP1*, *CDKN2A*, *SNCA*, *NR4A1*, *IL-6* and *FABP4*) were constructed and validated in training and testing cohorts. Kaplan-Meier curve and receiver operating characteristic (ROC) curve analysis confirmed that that the ferroptosis-related eight gene signature had good predictive value for the prognosis of thyroid cancer (THCA) patients. Multivariate regression analysis further showed that the risk score of the prognostic model could be used as an independent prognostic factor for THCA patients. Functional enrichment analysis showed that DEGs in high risk and low risk groups were involved in immune-related biological processes and that there were significant differences in immune cell infiltration between the two risk groups.

**Conclusion:**

We identified eight key genes related to ferroptosis in THCA patients. Further studies are now needed to investigate the mechanisms involved; these genes may represent clinical diagnostic and prognostic biomarkers.

## Introduction

Thyroid cancer (THCA) is the most common malignant neoplasm of the endocrine system and the incidence of this disease is increasing in many countries and regions (1). Thyroid follicular epithelial cell-derived cancers represent over 90% of all thyroid cancers and are divided into papillary thyroid cancer (PTC; approximately 90%), follicular thyroid cancer (FTC; approximately 10%) and anaplastic thyroid cancer (ATC, < 2%). Medullary thyroid cancer (MTC) represents less than 5% of all thyroid cancers (2). Thyroid malignancies are stratified by genetic background and aggressiveness in the latest world health organization (WHO) classification, in which PTC stands for BRAF-like malignancies, while invasive encapsulated follicular variant papillary carcinoma and follicular-derived carcinoma represent RAS-like malignancies, thus emphasizing the importance of genetic background to tumor biology (3). Although most thyroid cancers have a good and predictable prognosis, anaplastic, medullary and refractory thyroid cancers are still prone to recurrence and metastasis, thus resulting in a poor prognosis (4). At present, many targeted therapies are used clinical practice, including sorafenib, a small molecule tyrosine kinase inhibitor that targets *VEGFR*, *BRAF* and *RET*, to treat advanced or metastasized thyroid cancer. However, the efficacy of sorafenib is limited due to its association with adverse events such as hand foot syndrome (HFS) (5). Therefore, there is an urgent need to discover novel and reliable genes to judge tumor aggressiveness and biological behavior and to provide important guidance for the precise treatment of THCA patients with a poor prognosis.

Ferroptosis is a novel modality of regulated cell death (RCD) that, unlike other RCDs (such as apoptosis, autophagic cell death, necroptosis and pyroptosis), mainly depends on iron-mediated lipid peroxidation and cell membrane damage (6). In recent years, it has been found that ferroptosis was related to the occurrence and treatment response of various types of tumors (7). Furthermore, studies have shown that ferroptosis was associated with thyroid cancer. Vitamin C induces ferroptosis in ATC cells by promoting ROS generation, the activation of ferritin phagocytosis and the accumulation of iron (8). The knockdown of *ETV4* has been shown to downregulate *SLC7A11*, which in turn promoted ferroptosis to inhibit PTC development (9). However, research relating to the role and mechanisms of ferroptosis in thyroid cancer is very limited.

The tumor microenvironment (TME) is a complex ecosystem that includes not only the tumor cells themselves, but also immune cells, fibroblasts, angiogenic vascular cells (AVC), endothelial cells, glial cells, smooth muscle cells, epithelial cells, fat cells and other cellular components. The TME also contains non-cellular components such as extracellular matrix (ECM), cytokines, chemokines, growth factors and antibodies (10, 11). Interactions between the various components of the TME are significant. Tumor cells can change the nature of this microenvironment, and conversely, the microenvironment can affect how a tumor grows and spreads. Thus, the TME plays a key role in regulating immune responses in cancer patients. Numerous studies have shown that ferroptosis has dual tumor-promoting and tumor-inhibiting roles during tumorigenesis, which depend on the release of damage-associated molecular patterns (DAMPs) in the TME and injury caused by ferroptosis, thus triggering activation of the immune response (12). In addition, ferroptosis affects the efficacy of chemotherapy, radiotherapy and immunotherapy; therefore, combinations with agents targeting ferroptosis signaling could improve the outcomes of such therapies.

In this study, we constructed a prognostic risk model based on eight FRGs and validated its prognostic efficacy. We also performed functional enrichment analysis to explore the potential mechanisms involved. In addition, we assessed the correlation of risk models with immune cell infiltration. Our findings helped to elucidate the role of FRGs in THCA and provided new therapeutic targets for improving the prognosis of patients with thyroid cancer.

## Materials and methods

### Data collection and definition of ferroptosis-related genes

THCA mRNA expression profiles and corresponding clinical data were obtained from the UCSC Xena browser (https://xenabrowser.net/datapages/), including 497 tumor samples and 56 normal tissue samples. All data from TCGA are publicly available. This study was exempted from local ethics committee approval and complied with the TCGA Data Access Policy and Publication Guidelines.

In total, 567 ferroptosis-related genes (FRGs) were identified from the ferroptosis database (http://www.zhounan.org/ferrdb/current/), including 369 ferroptosis drivers, 348 ferroptosis suppressors, 11 ferroptosis markers and 116 unclassified genes (**Supplementary Table 1**). We further removed duplicated genes in four subgroups of the ferroptosis genome, thus resulting in a total of 567 genes for subsequent analysis.

### Identification and confirmation of ferroptosis-related genes in THCA

Differentially expressed genes (DEGs) in tumor tissues and adjacent normal tissues in the TCGA THCA cohort were analyzed by the DESeq2 package in R (version 4.2.0; https://cran.r-project.org/). Genes that with a (|log_2_FC|> 1 and p < 0.05 were considered significantly different. The R venn package was used to identify crossover genes between DEGs and FRGs to obtain ferroptosis-related DEGs in THCA.

### Construction and validation of a ferroptosis-related prognosis signature

Univariate Cox regression analysis was used to screen for DEGs associated with the prognosis of patients with THCA; those with p < 0.05 were statistically significant. The R venn package was used to identify crossover genes between ferroptosis-related DEGs and prognosis-related DEGs to obtain potential prognosis-related FRGs.

The STRING database (http://string-db.org) was used to predict the protein-protein interactions (PPI) of prognosis-related FRGs and to construct PPI networks with a minimum required interaction score ≥0.15. Further analysis was performed with cytoscape software and the cytoHubba plugin was used to identify central genes by degree order; the top 10 hub genes were selected for subsequent analysis.

LASSO-Cox regression was performed using ten prognosis-related FRGs based on the training cohort (n = 497 for the entire data set) to select the best prognostic genes. Using the glmnet package in R, cox was selected as the family and 10-fold cross-validation and 1,000 iterations were performed to select the optimal value of the penalty parameter (λ) and determine the genes to be included in the model.

Subsequently, we extracted the Cox multivariate regression coefficient for each prognostic gene and the gene expression level was used to calculate the risk score by the following formula: risk score=βmRNA1×expr mRNA1+βmRNA2×expr mRNA2+⋯+βmRNAn×expr mRNAn, where βmRNAn represents the Cox hazard proportionality coefficient of mRNAn and expr mRNA represents the expression level of the gene. The risk score for each patient in both the training cohort and the testing cohort (randomly selected, n =248) was estimated based on the formula and patients were classified into high- and low-risk groups stratified by the median risk score. Differences in survival between the high- and low-risk groups were analyzed by Kaplan-Meier curves. The ROC package and the time ROC package in R were used to draw receiver operating characteristic (ROC) curves and calculate the area under the curve (AUC), predict overall survival, 1-year, 3-year and 5-year survival and to evaluate the prognostic value of the FRG-related risk model for both the training cohort and the testing cohort.

### Construction of a nomogram between the prognostic risk model and clinicopathological factors in thyroid cancer

To further evaluate the predictive power of the risk score model, a nomogram was constructed using the rms package in R by combining the other clinicopathological characteristics of thyroid cancer patients; we then analyzed the factors affecting survival. We also calculated the consistency index (C-index) of the nomogram to evaluate its predictive accuracy; the closer the C-index was to 1 (the value range is 0 - 1), the greater the predictive value of the constructed regression model. In addition, the bootstrap resampling method was used to construct a nomogram calibration plot for the internal sample to verify the consistency of the survival rate predicted by the model with the actual survival rate.

### Functional enrichment analysis

DEGs between high and low risk groups were identified using the DESeq2 package in R (|log_2_FC|>1, p<0.05). The clusterProfiler package was used for Gene Ontology (GO) and Kyoto Encyclopedia of Genes and Genomes (KEGG) analysis, with p < 0.05, a multiple hypothesis testing p value corrected by the Benjamini and Hochberg method and a q value < 0.05. The GO database divides the functions of genes into three aspects: cellular component (CC), molecular function (MF) and biological process (BP). The KEGG database, in addition to annotation of the function of the gene itself, integrates genomic, chemical and systemic functional information involving many signaling pathways, diseases and drugs, and has become a comprehensive database for the functional interpretation and practical application of genomic information.

### Immune-related analysis

The stromal score (stromal content), immune score (degree of immune cell infiltration) and ESTIMATE score (a composite marker of stroma and immunity) were obtained for each tumor sample by evaluating the immune microenvironment of each tumor sample using the estimate package in R (13). The weighted gene co-expression network analysis (WGCNA) was used to perform modular analysis of DEGs. In brief, the scale-free topology criterion was used to calculate the soft threshold. The optimal soft threshold was chosen and the minimum module size was set to 30 genes. Then, we used the dynamic tree cut recognition module and set the MEDissThres parameter to 0.25. After correlating each module with the immune microenvironment score, the modules with a Pearson’s correlation coefficient > 0.5 were selected as target modules. The DEGs in the target module and the differential fold of these genes were then imported into R and KEGG analysis was performed using the clusterProfiler package.

The proportions of 22 immune cells in each tumor sample were calculated based on expression profiles using the CIBERSORT package in R, and the sum of the fractions of the 22 immune cell types in each sample was 1 (14). The correlations between the eight FRGs in the risk model and immune cells were analyzed by Pearson’s correlation using CIBERSORT.

By applying the Single-Sample Gene Set Enrichment Analysis (ssGSEA) method of the GSVA package in R, the degree of infiltration for 28 immune cell types was calculated based on the gene expression levels in the 28 immune cell gene sets (15). The differences in immune cell infiltration between the two groups were analyzed according to the grouping information for the high-risk group and the low-risk group.

### Statistical analysis

All statistical analyses were performed using R software(version 4.2.0). Comparisons of the tumorous and normal tissues were performed by the Wilcoxon test. Kaplan-Meier curves and log-rank tests were used to compare the OS between high and low risk groups. Univariate and multivariate Cox regression analyses were used to determine independent predictors of OS. Comparisons of ssGSEA scores for immune cells and pathways between high and low risk groups was performed with Wilcoxon’s test. P values < 0.05 were considered statistically significant.

## Results

**Figure 1:**
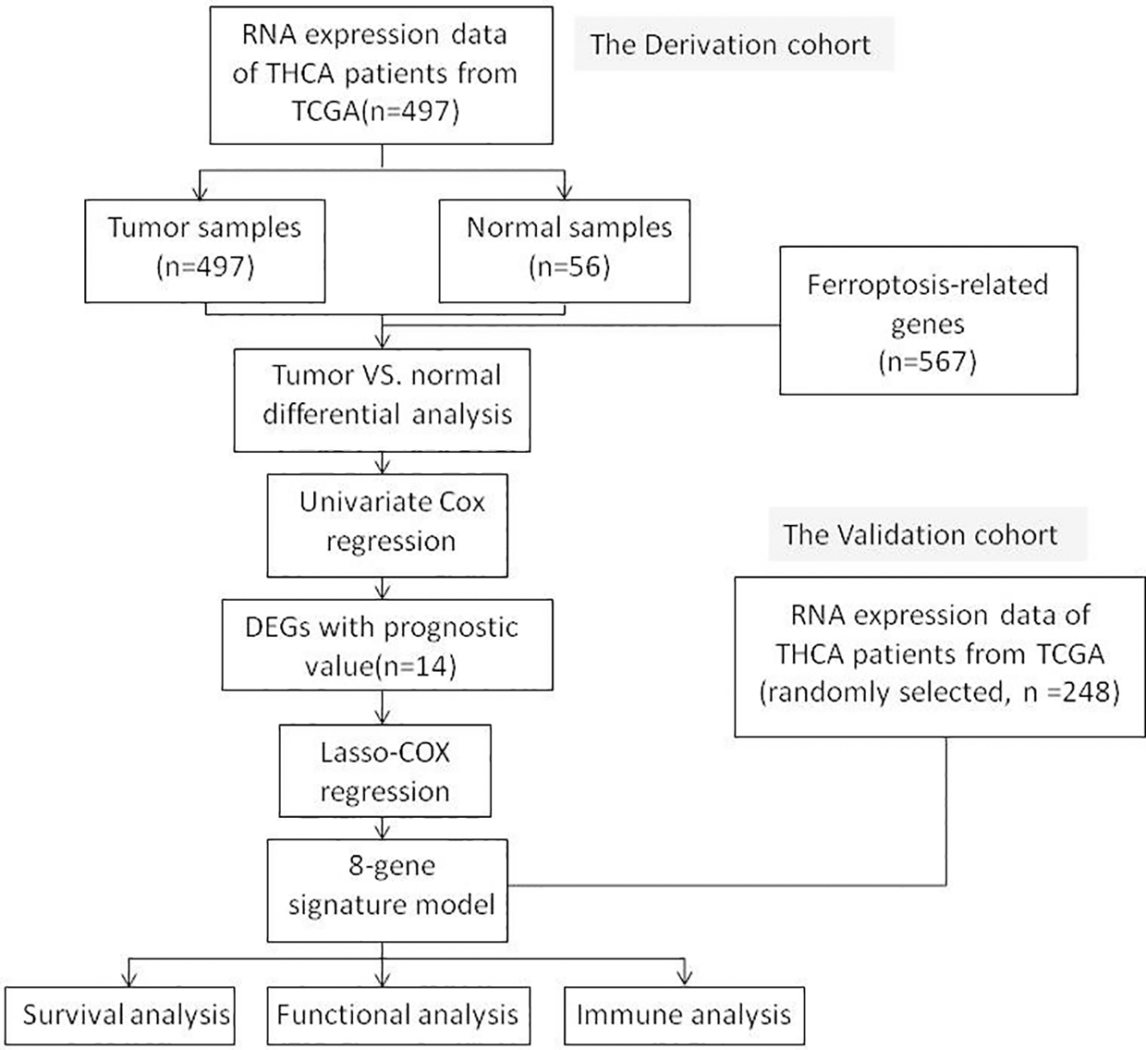
A flowchart depicting the construction and validation of data collection and analysis.

**Figure 1** shows a flowchart depicting the construction and validation of data collection and analysis. The baseline clinical characteristics of the thyroid cancer patients in this study are summarized in **Table 1**.

**Table 1:** The baseline clinical characteristics of the thyroid cancer patients in this study.

	TCGA cohort
No. of patients	496*
Age(median, range)	46 (15-89)
Stage (%)	
I	279
II	52
III	110
IV	53
Unknown	2
T	
1	141
2	162
3	169
4	22
Unknown	2
N	
0	227
1	220
Unknown	49
M	
0	282
1	8
Unknown	206
Survival status	
OS days (median)	944

^*^Clinical information was missing for one of the patients in this study.

### Identification of prognostic ferroptosis-related DEGs in the TCGA cohort

**Figure 2:**
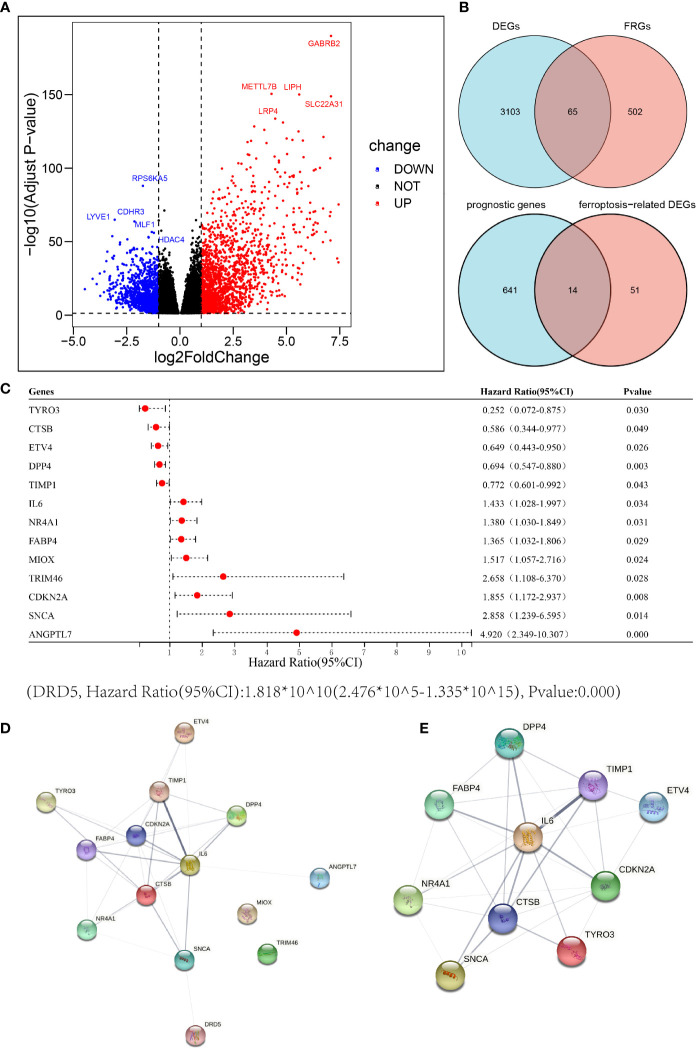
Identification of candidate ferroptosis-related genes in the cancer genome atlas (TCGA) cohort. **(A)** Differentially expressed genes between THCA and adjacent normal thyroid tissue are shown by a volcano plot. **(B)** Venn diagrams to identify differentially expressed ferroptosis-related genes between tumor and adjacent normal tissue that were correlated with OS. **(C)** Univariate Cox regression analysis between gene expression and OS are shown by a Forest plot. **(D, E)** The PPI network downloaded from the STRING database indicated the interactions among the candidate genes.

In the TCGA THCA cohort, we identified a total of 3168 differentially expressed genes (DEGs) in tumor tissues and adjacent normal tissues; of these, 1,857 were up-regulated in tumor tissues and 1,311 were down-regulated (**Figure 2A**). The top five up-regulated genes were *GABRB2*, *METTL7B*, *LIPH*, *SLC22A31* and *LRP4*. The top five down-regulated genes were *RPS6KA5*, *LYVE1*, *CDHR3*, *MLF1* and *HDAC4*. We included a total of 567 well-defined FRGs in this study and obtained 65 differentially expressed FRGs by intersecting these 567 FRGs with 3,168 DEGs, thus indicating that 65 FRGs were differentially expressed in thyroid cancer tumor tissue and adjacent normal tissue. Fourteen of these FRGs were significantly associated with overall survival (OS) in univariate Cox regression analysis (**Figure 2B**). Thus, a total of 14 prognostic ferroptosis-related DEGs were identified. The forest plot shown in **Figure 2C** depicts the results of the univariate Cox regression analysis for these 14 genes. The results showed that five of these genes with a hazard ratio (HR) of < 1 played a protective role in THCA patients (*ETV4*, *DPP4*, *TYRO3*, *TIMP1* and *CTSB*) while the other 9 genes (*CDKN2A*, *TRIM46*, *SNCA*, *NR4A1*, *MIOX*, *IL-6*, *FABP4*, *ANGPTL7* and *DRD5*) were risk factors with a HR > 1. The PPI network provided interactive information between these 14 differentially expressed prognostic FRGs (**Figure 2D**). The hub genes were analyzed using cytoscape and the top 10 (*IL-6*, *CDKN2A*, *CTSB*, *TIMP1*, *FABP4*, *NR4A1*, *DPP4*, *SNCA*, *TYRO3* and *ETV4*) were selected for subsequent analysis (**Figure 2E**).

### Construction and validation of A 8−FRG signature predicting the prognosis of THCA

**Figure 3:**
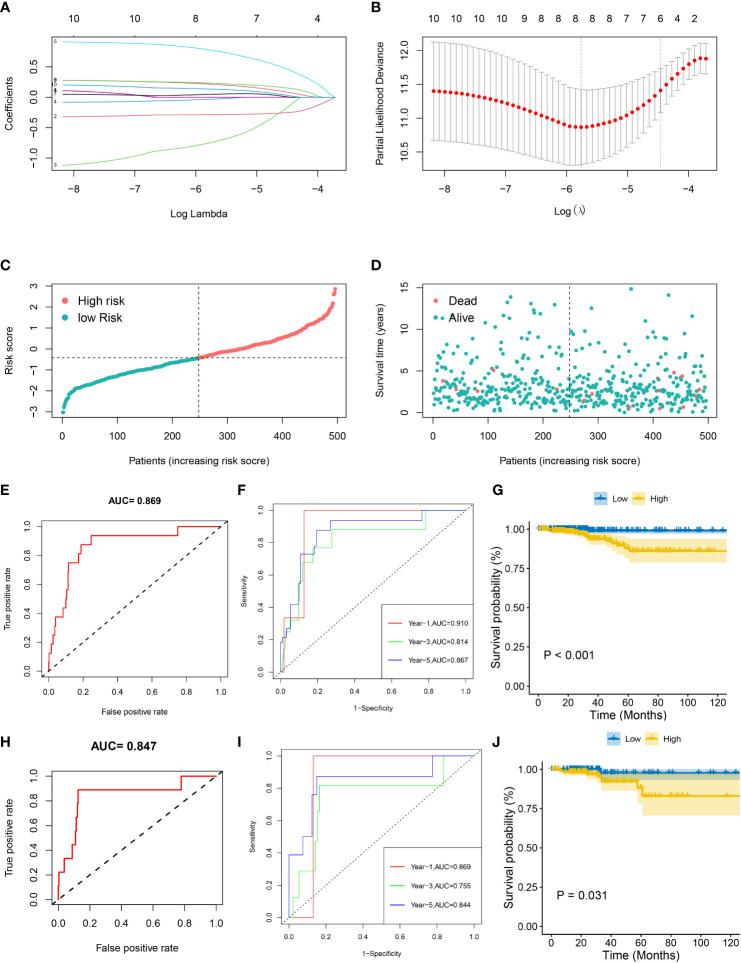
Prognostic analysis of the eight gene signature model in the TCGA cohort. **(A)** Tenfold cross-validation for tuning parameter selection in the LASSO model. **(B)** LASSO coefficient profiles of the eight prognostic genes for THCA. **(C, D)** The distribution and median value of the risk scores in the TCGA cohort. **(E, F)** The AUC ROC curves verified the prognostic performance of the risk score in the TCGA training cohort. **(H, I)** The AUCs of ROC curves verified the prognostic performance of the risk score in the TCGA testing cohort. **(G, J)** Kaplan-Meier curves for the OS of patients in the high-risk group and low-risk group in both the TCGA training and testing cohorts.

The 10 FRGs related to prognosis were substituted into a Lasso-Cox regression model and the optimal lambda value was selected as 0.003 (**Figures 3A, B**). Eight FRGs were finally identified and used to construct a prognostic risk model; the eight genes were *DPP4*, *TYRO3*, *TIMP1*, *CDKN2A*, *SNCA*, *NR4A1*, *IL-6*, *FABP4*. Then, we constructed a THCA prognostic model risk score based on the eight FRGs as follows: *DPP4* × (-0.287) + *TYRO3* ×(-0.747) + *TIMP1* × (-0.033) + *CDKN2A* ×0.775 + *SNCA* ×0.047 + *NR4A1* ×0.215 + *IL-6* ×0.231 + *FABP4* × 0.134. Patients were divided into high and low risk groups according to the median risk score of -0.422. The risk map distribution and survival status of THCA patients showed that the OS rate of patients in the high risk group was significantly lower (p < 0.05) than that of the patients in the low risk group (**Figures 3C, D**).

ROC and Kaplan-Meier curves were used to assess the prognostic value of the eight-gene model in the training cohort (the whole dataset, n = 497) and the testing cohort (randomly selected, n = 248). Kaplan-Meier survival curves confirmed that the OS of patients in the high-risk group was significantly lower than that in the low risk group in both the training (p < 0.001) and testing (p < 0.05) cohorts (**Figures 3G, J**). In the training cohort, the AUC of the ROC curve was 0.869 while the AUCs of the time-dependent ROC curves at 1 year, 3 years, and 5 years were 0.910, 0.814 and 0.866, respectively (**Figures 3E, F**). In the testing cohort, the AUC of the ROC curve was 0.847 while the AUCs of the time-dependent ROC curves at 1, 3, and 5 years were 0.869, 0.755, and 0.844, respectively (**Figures 3H, I**). These results suggested that the eight gene prognostic risk model performs well in terms of survival prediction.

**Figure 4:**
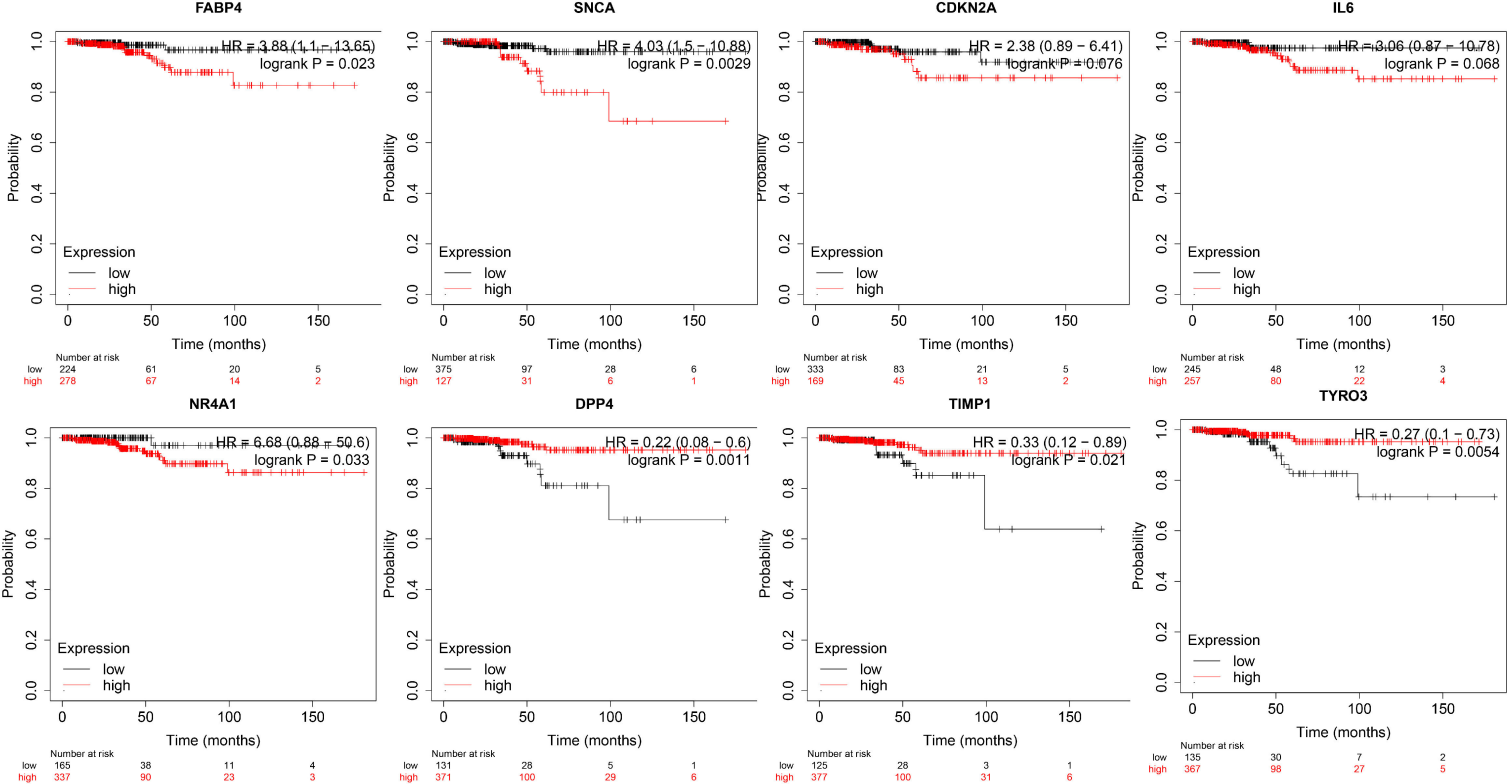
Kaplan-Meier curves of genes associated with the eight gene prognostic risk signature.

Based on the TCGA Thyroid Cancer Database, we further performed survival analysis on the eight genes in the prognostic risk model. We found that *DPP4*, *TIMP1* and *TYRO3* were associated with a better prognosis and survival in patients with thyroid cancer, while *FABP4*, *NR4A1* and *SNCA* were associated with a poor prognosis and survival in patients with thyroid cancer (**Figure 4**).

### Associations of the risk model with overall survival and the clinicopathological characteristics of patients with thyroid cancer

**Figure 5:**
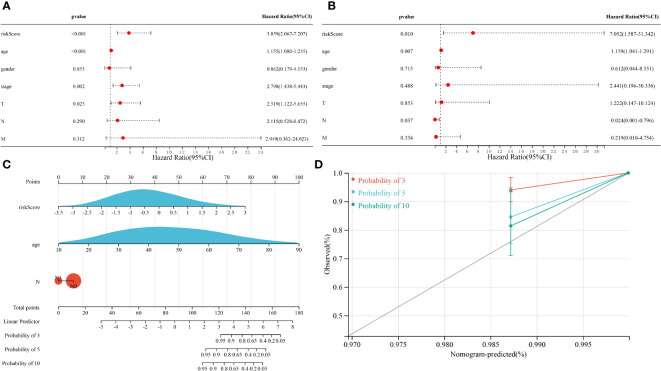
Results of the Cox regression analyses regarding OS in the TCGA cohort. **(A)** Univariate Cox regression analyses regarding OS in the TCGA cohort. **(B)** Multivariate Cox analyses regarding OS in the TCGA cohort. **(C)** A nomogram constructed from prognostic risk scores and clinicopathological factors. **(D)** Calibration curve for the nomogram.

Univariate Cox regression analysis showed that risk score, age, tumor stage and pathological T stage were significantly associated with OS in patients with THCA (p < 0.05, **Figure 5A**). Multivariate Cox regression analysis showed that risk score, age and pathological N stage were significantly associated with OS in THCA patients (p < 0.05, **Figure 5B**). These results suggested that the risk score of the risk model could serve as an independent prognostic factor for patients with THCA. To provide THCA prognosis by composite risk score, age, and pathological N stage, we established a quantitative nomogram to predict patient-individualized survival time. As shown in **Figure 5C**, age and the risk score of the prognostic risk model had a greater impact on the predictive ability of the nomogram; the older the age, the lower the survival rate, and the higher the risk score of the prognostic risk model, the lower the survival rate. In addition, the C-index of the nomogram was 0.960 (p < 0.001), showing good agreement between predicted 3-, 5-, and 10-year survival and actual survival (**Figure 5D**).

### Functional analysis of biological pathways associated with the risk model

**Figure 6:**
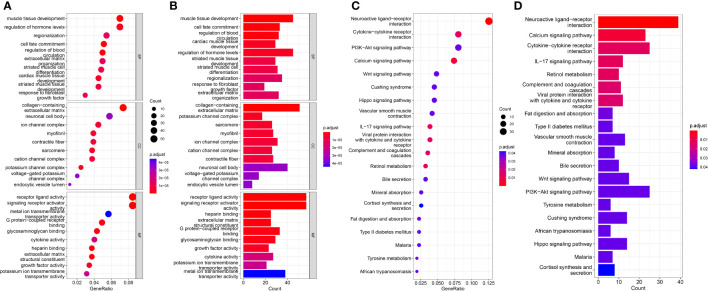
Results of GO and KEGG analyses in the TCGA cohort. **(A, B)** Significant GO enrichment in the TCGA cohort. **(C, D)** Significant KEGG pathways in the TCGA cohort.

DEGs for the high risk and low risk groups were analyzed and a total of 758 genes were obtained. To elucidate the underlying biological functions and pathways associated with our eight-gene prognostic signature model, we performed GO enrichment and KEGG pathway analysis of DEGs between the high and low risk groups. Interestingly, the results showed that these DEGs were significantly enriched in many TME-related biological processes in GO, including response to fibroblast growth factor, extracellular matrix organization, collagen-containing extracellular matrix, endocytic vesicle lumen, signaling receptor activator activity, extracellular matrix structural constituent, growth factor activity and cytokine activity (**Figures 6A, B**). KEGG analysis revealed enrichment in some TME-related pathways, such as cytokine-cytokine receptor interaction, the *IL-17* signaling pathway, the Wnt signaling pathway and the PI3K-Akt signaling pathway (**Figures 6C, D**).

### Risk model-related immune microenvironment analysis

**Figure 7:**
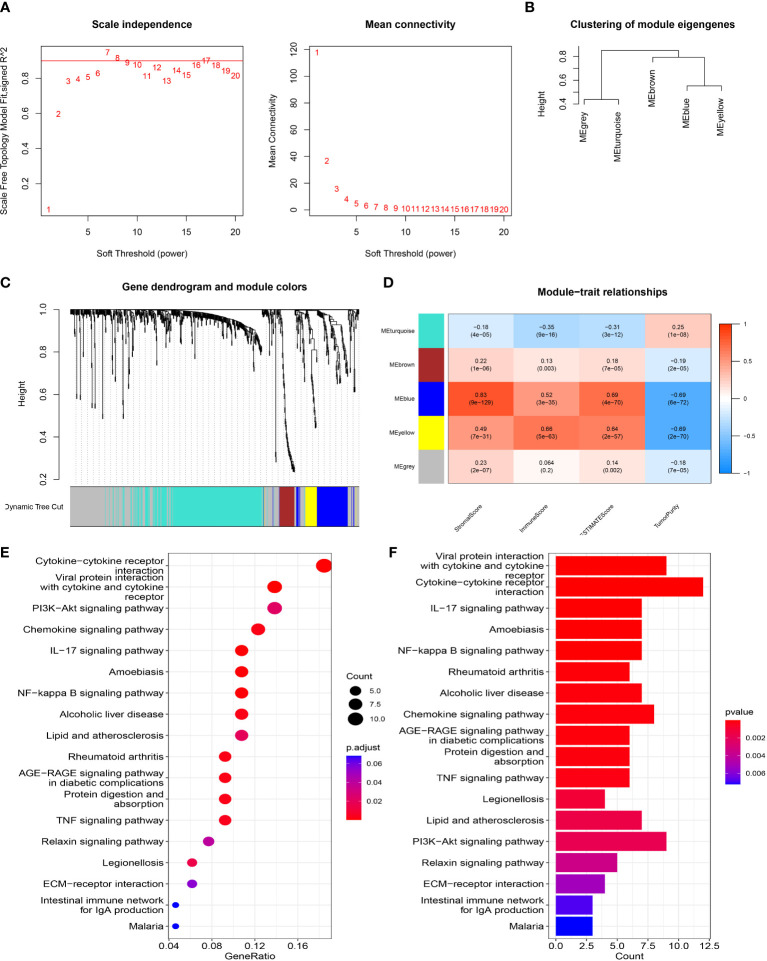
WGCNA network module mining. **(A)** We determined the best soft threshold by network topology analysis. **(B)** Hierarchical clustering analysis of WGCNA modules. **(C)** Gene dendrogram and nodule color of WGCNA. **(D)** The correlation between modules and immune microenvironment scores. **(E, F)** KEGG analysis.

To elucidate the relationship between the eight-gene prognostic signature model and the immune microenvironment, we assessed the immune microenvironment of each tumor sample and determined the stromal score, immune score and composite score for tumor samples. The correlation between DEGs in the high and low risk groups and immune scores was then analyzed by WGCNA. First, we computed the soft threshold using scale-free topological criteria. When the soft threshold power β was seven, the connectivity between genes in the gene network satisfied the scale-free network distribution (**Figure 7A**). Then, co-expression modules were mined using a phylogenetic tree. Modules were analyzed by hierarchical clustering and modules on the same branch showed similar gene expression patterns, obtaining five co-expression modules (95 in blue, 39 in brown, 274 in grey, 318 in cyan and 32 in yellow) (**Figures 7B, C**). Subsequently, gene clusters were visualized and the correlation of modules with immune microenvironment scores was analyzed. Analysis showed that the blue module had a higher correlation with the stromal score while the yellow and blue modules also had a good correlation with the immune score (**Figure 7D**). We further performed KEGG analysis on the 127 DEGs in the blue and yellow modules and found that these DEGs were significantly enriched in many immune-related pathways in KEGG analysis, including the *IL-17* signaling pathway, NF-kappa B signaling pathway, TNF signaling pathway and *PI3K-Akt* signaling pathway (**Figures 7E, F**). These results suggested that our eight gene prognostic model was highly correlated with immunity.

### Risk model-related infiltrating immune cells analysis

**Figure 8:**
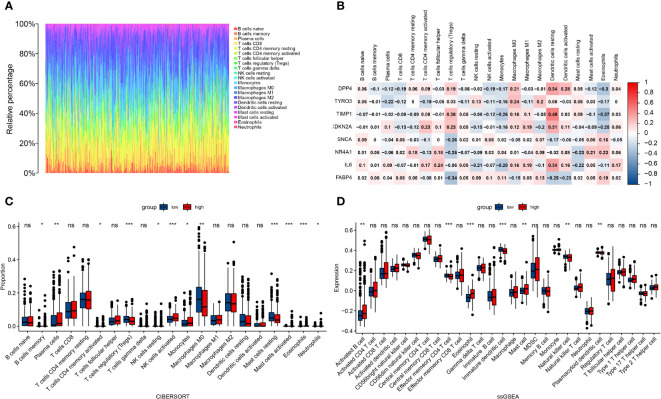
Correlation between the risk score of the prognostic risk model and immune cells. **(A)** Immune cell proportions for each tumor patient. **(B)** The correlation between eight FRGs and immune cells. **(C)** The infiltration levels of 22 immune cell subtypes in the high and low risk groups. **(D)** Quantification of distinct immune cell subsets by ssGSEA.*: p < 0.05, ;** : p < 0.01, ***: p < 0.001. ns: no significance.

To further explore the correlation between the risk score of the prognostic risk model and immune cells, we calculated various immune cell proportions for each tumor patient with CIBERSORT (**Figure 8A**). As we expected, many immune cells showed statistical differences between the high and low risk groups in the TCGA cohort, including B cell memory, plasma cells, T cells CD4 memory (activated), T cell regulatory (Tregs), NK cells (resting), NK cells (activated), monocytes, macrophages M0, mast cells (resting), Mast cells (activated), eosinophils and neutrophils (**Figure 8C**). Next, we evaluated the correlation between the eight FRGs and immune cells and found that *TIMP1* had a good correlation with dendritic and eosinophils. *FABP4* was negatively correlated with Tregs; *IL-6* and *DPP4* were also correlated with Dendritic cells (resting) (**Figure 8B**). Subsequently, we quantified different immune cell subsets by ssGSEA. The results showed that the numbers of effector memory CD4 T cells, immature dendritic cells, natural killer cells and plasmacytoid dendritic cells in the TME of the high risk group were significantly lower than those of the low risk group, while activated B cells, eosinophil and mast cells were more abundant in the high risk group (**Figure 8D**).

## Discussion

As the most common malignant tumor of the endocrine system, the incidence of THCA is increasing; furthermore, the mortality of advanced thyroid cancer and ATC has also increased. Ferroptosis has been shown to be involved in many forms of cancer, such as non-small cell lung cancer, breast cancer, pancreatic cancer, and hepatocellular carcinoma (16). Ferroptosis induction recently emerged as an attractive strategy for cancer therapy. Studying the mechanism of ferroptosis in THCA may help us identify more appropriate and effective therapeutic targets to improve survival.

Previous studies found that ferroptosis regulated the progression of THCA (8, 9, 17). Consistent with previous findings, we found that 65 ferroptosis-related genes were differentially expressed between thyroid cancer tumor tissues and adjacent non-tumor tissues, of which 14 were significantly associated with OS. These results fully demonstrate the important role of ferroptosis in thyroid cancer and the possibility of using ferroptosis-related genes to build prognostic models. We screened the top 10 key genes from 14 ferroptosis-related genes associated with thyroid cancer prognosis by PPI protein interaction network analysis. Finally, eight genes were screened by LASSO Cox regression analysis to construct a prognostic model, including *DPP4*, *TYRO3*, *TIMP1*, *CDKN2A*, *SNCA*, *NR4A1*, *IL-6* and *FABP4*. Of these, *DPP4*, *TIMP1*, *CDKN2A* and *SNCA* are genes that promote ferroptosis, *TYRO3*, *NR4A1* and *FABP4* are genes that inhibit ferroptosis while *IL-6* can promote or inhibit ferroptosis in different diseases. It is worth noting that in our study, the survival curves and ROC curves of the training group and the testing group both proved that the eight gene prognostic risk model performed well in the prediction of survival for patients with thyroid cancer. Multivariate regression analysis further showed that the risk score of the prognostic model is an independent prognostic factor for THCA patients. A literature search revealed that tumor suppressor p53 could inhibit erastin-induced ferroptosis by blocking *DPP4* activity (18). In addition, lncRNA AAB was found to sponge and sequester miR-30b-5p to induce the imbalance of *MMP9/TIMP1*, thus enhancing the activation of transferrin receptor 1 (TFR-1) and then eventually led to the ferroptosis of cardiac microvascular endothelial cells (CMECs) (19). The homozygous deletion of *CDKN2A/2B* has been identified as one of the major target genes involved in iron overload-induced carcinogenesis (20). Furthermore, in human iPSC-derived neurons with *SNCA* triplication, neuronal ferroptosis was induced due to the incorporation of excess α-synuclein oligomers into membranes (21). In contrast, *TYRO3*, *NR4A1* and *FABP4* are genes that suppress ferroptosis. The inhibition of *TYRO3* promoted tumor ferroptosis and sensitized resistant tumors to anti-programmed cell death protein 1 therapy (22). *NR4A1* inhibits ferroptosis and apoptosis by promoting the expression of stearoyl-CoA desaturase (*SCD1*) (23). A previous study found that *FABP4* was upregulated in recurrent human breast cancer samples; *FABP4* protects cancer cells from oxidative stress-induced ferroptosis and is associated with a worse prognosis in cancer patients (24). *IL-6* reversed ferroptosis and growth inhibition induced by xCT knockdown or erastin in head and neck squamous cell carcinoma (HNSCC) (25) but promoted ferroptosis in bronchial and mammary epithelial cells (26, 27). The association of these eight genes with ferroptosis has been clearly validated in other tumors and diseases; these associations will be the focus of future research on the relationship between THCA and ferroptosis.

As the internal environment for tumor cell generation and survival, the TME has been the focus of an increasing number of studies with regards to its influence on tumorigenesis, progression and metastasis (28). The TME, and especially immune infiltration, plays a key role in regulating immune responses in cancer patients (11). In our study, we performed functional analysis of DEGs between high risk and low risk groups based on the risk score of our eight gene prognostic model. Interestingly, we identified some biological processes related to the TME in GO and KEGG enrichment analyses, such as response to fibroblast growth factor, extracellular matrix organization, collagen-containing extracellular matrix, endocytic vesicle lumen, signaling receptor activator activity, extracellular matrix structural constituent, growth factor activity, cytokine activity, cytokine-cytokine receptor interaction and the *IL-17* signaling pathway, thus suggesting that our prognostic model may be closely related to the TME of thyroid cancer. Therefore, we further analyzed the correlation between DEGs in high risk and low risk groups and the TME and found that 127 genes were highly correlated with immune score. KEGG analysis of these genes further confirmed that they were significantly enriched in immune-related pathways, including the *IL-17* signaling pathway, NF-kappa B signaling pathway and TNF signaling pathway. Given the important role of immune cell infiltration in the immune response of tumor patients, we investigated differences in immune cell infiltration between high-risk and low-risk groups. The results showed that the infiltration degree of effect memory CD4 T cells, immature dendritic cells, plasmacytoid dendritic cells and natural killer cells in the high-risk group of thyroid cancer patients decreased, while the infiltration degree of activated B cells, eosinophils and mast cells increased, thus indicating that our eight-gene prognostic model may be a predictor of immune responses in thyroid cancer. Numerous studies have shown that NK cells played a key role in anti-tumor activities (29). Immature dendritic cells, despite being functionally defective, are effective vehicles for immunotherapy using DC/tumor cell fusion vaccines (30). This is consistent with our results in natural killer cells and immature dendritic cells, in that the numbers of these anti-tumor immune cells were reduced in the high-risk group of patients. A previous study found that Mast cells had a pro-tumorigenic role in human thyroid cancer (31); this is consistent with our observation of an increased number of mast cells in our high risk group. Eosinophils play different roles in different tumors; for example, eosinophils have anti-tumor effects in colon cancer but exert tumor-promoting activity in primary breast cancer (32). In our study, the number of eosinophils was higher in the high risk group of patients with thyroid cancer. Plasmacytoid DCs (pDCs) are one of two major subpopulations of human dendritic cells. Our past understanding of pDC biology is that they are specialized effectors of anti-viral and anti-tumor immunity. However, increasing evidence suggests that pDC infiltration into the tumor microenvironment is associated with tumor development and a poor prognosis (33). The infiltration level of pDCs in the high risk group in our model was reduced; therefore, further studies of the prognostic significance of pDCs in patients with thyroid cancer is needed. Taken together, our data indicated that imbalanced immune infiltration and the dysfunction of immune responses in the TME may be attributable to high risk scores, at least in part.

Inevitably, our study has certain limitations that need to be considered. We successfully revealed that the risk score of the eight gene risk model could be used as an independent prognostic factor for thyroid cancer patients through comprehensive bioinformatics analysis and analyzed the relationship between the risk score and the level of immune cell infiltration. However, further *in vitro* and *in vivo* experiments are needed to confirm these conclusions.

In conclusion, we constructed a novel ferroptosis-related gene prognostic model consisting of eight FRGs and risk scores and independently predicted the prognosis of thyroid cancer patients. New ferroptosis-related genes may be used in thyroid cancer targeted therapy in the future.

## Data availability statement

Publicly available datasets were analyzed in this study. This data can be found here: https://xenabrowser.net/datapages/.

## Author contributions

XR and HD contributed equally to this work. All authors listed have made a substantial, direct, and intellectual contribution to the work, and approved it for publication.

## Funding

This research was supported by Research project supported by China Health Promotion Foundation[CDPF-2017-LXSCD].

## Conflict of interest

The authors declare that the research was conducted in the absence of any commercial or financial relationships that could be construed as a potential conflict of interest.

## Publisher’s note

All claims expressed in this article are solely those of the authors and do not necessarily represent those of their affiliated organizations, or those of the publisher, the editors and the reviewers. Any product that may be evaluated in this article, or claim that may be made by its manufacturer, is not guaranteed or endorsed by the publisher.

## References

[1] Pizzato M Li M Vignat J Laversanne M Singh D La Vecchia C. The epidemiological landscape of thyroid cancer worldwide: GLOBOCAN estimates for incidence and mortality rates in 2020. Lancet Diabetes Endocrinol (2022) 10(4):264–72. doi: 10.1016/S2213-8587(22)00035-3 35271818

[2] Fallahi P Ferrari SM Galdiero MR Varricchi G Elia G Ragusa F. Molecular targets of tyrosine kinase inhibitors in thyroid cancer. Semin Cancer Biol (2022) 79:180–96. doi: 10.1016/j.semcancer.2020.11.013 33249201

[3] Baloch ZW Asa SL Barletta JA Ghossein RA Juhlin CC Jung CK. Overview of the 2022 WHO classification of thyroid neoplasms. Endocr Pathol (2022) 33(1):27–63. doi: 10.1007/s12022-022-09707-3 35288841

[4] Liu Q Sun W Zhang H. Roles and new insights of macrophages in the tumor microenvironment of thyroid cancer. Front Pharmacol (2022) 13:875384. doi: 10.3389/fphar.2022.875384 35479325 PMC9035491

[5] Puerto M Borson-Chazot F Tabarin A. Updates on therapy for medullary thyroid cancer in 2021. Ann Endocrinol (Paris) (2022) 83(2):114–8. doi: 10.1016/j.ando.2021.12.002 34921811

[6] Jiang X Stockwell BR Conrad M. Ferroptosis: Mechanisms, biology and role in disease. Nat Rev Mol Cell Biol (2021) 22(4):266–82. doi: 10.1038/s41580-020-00324-8 PMC8142022 33495651

[7] Zhang C Liu X Jin S Chen Y Guo R. Ferroptosis in cancer therapy: A novel approach to reversing drug resistance. Mol Cancer (2022) 21(1):47. doi: 10.1186/s12943-022-01530-y 35151318 PMC8840702

[8] Wang X Xu S Zhang L Cheng X Yu H Bao J. Vitamin c induces ferroptosis in anaplastic thyroid cancer cells by ferritinophagy activation. Biochem Biophys Res Commun (2021) 551:46–53. doi: 10.1016/j.bbrc.2021.02.126 33714759

[9] Wang L Zhang Y Yang J Liu L Yao B Tian Z. The knockdown of ETV4 inhibits the papillary thyroid cancer development by promoting ferroptosis upon SLC7A11 downregulation. DNA Cell Biol (2021) 40(9):1211–21. doi: 10.1089/dna.2021.0216 34283663

[10] Dou A Fang J. Heterogeneous myeloid cells in tumors. Cancers (Basel) (2021) 13(15):3772. doi: 10.3390/cancers13153772 34359674 PMC8345207

[11] Peña-Romero AC Orenes-Piñero E. Dual effect of immune cells within tumour microenvironment: Pro- and anti-tumour effects and their triggers. Cancers (Basel) (2022) 14(7):1681. doi: 10.3390/cancers14071681 35406451 PMC8996887

[12] Chen X Kang R Kroemer G Tang D. Broadening horizons: The role of ferroptosis in cancer. Nat Rev Clin Oncol (2021) 18(5):280–96. doi: 10.1038/s41571-020-00462-0 33514910

[13] Yoshihara K Shahmoradgoli M Martínez E Vegesna R Kim H Torres-Garcia W. Inferring tumour purity and stromal and immune cell admixture from expression data. Nat Commun (2013) 4:2612. doi: 10.1038/ncomms3612 24113773 PMC3826632

[14] Newman AM Liu CL Green MR Gentles AJ Feng W Xu Y. Robust enumeration of cell subsets from tissue expression profiles. Nat Methods (2015) 12(5):453–7. doi: 10.1038/nmeth.3337 PMC4739640 25822800

[15] Hänzelmann S Castelo R Guinney J. GSVA: gene set variation analysis for microarray and RNA-seq data. BMC Bioinf (2013) 14:7. doi: 10.1186/1471-2105-14-7 PMC3618321 23323831

[16] Zhao L Zhou X Xie F Zhang L Yan H Huang J. Ferroptosis in cancer and cancer immunotherapy. Cancer Commun (Lond) (2022) 42(2):88–116. doi: 10.1002/cac2.12250 35133083 PMC8822596

[17] Ji FH Fu XH Li GQ He Q Qiu XG. FTO prevents thyroid cancer progression by SLC7A11 m6A methylation in a ferroptosis-dependent manner. Front Endocrinol (Lausanne) (2022) 13:857765. doi: 10.3389/fendo.2022.857765 35721711 PMC9205202

[18] Xie Y Zhu S Song X Sun X Fan Y Liu J. The tumor suppressor p53 limits ferroptosis by blocking DPP4 activity. Cell Rep (2017) 20(7):1692–704. doi: 10.1016/j.celrep.2017.07.055 28813679

[19] Shi P Li M Song C. Neutrophil-like cell membrane-coated siRNA of lncRNA AABR07017145.1 therapy for cardiac hypertrophy *via* inhibiting ferroptosis of CMECs. Mol Ther Nucleic Acids (2022) 27:16–36. doi: 10.1016/j.omtn.2021.10.024 34938604 PMC8646082

[20] Toyokuni S. Mysterious link between iron overload and CDKN2A/2B. J Clin Biochem Nutr (2011) 48(1):46–9. doi: 10.3164/jcbn.11-001FR PMC3022063 21297911

[21] Angelova PR Choi ML Berezhnov AV Horrocks M H Hughes C D De S. Alpha synuclein aggregation drives ferroptosis: an interplay of iron, calcium and lipid peroxidation[J]. Cell Death Differ (2020) 27(10):2781–96. doi: 10.1038/s41418-020-0542-z PMC7492459 32341450

[22] Jiang Z Lim SO Yan M Hsu JL Yao J Wei Y. TYRO3 induces anti-PD-1/PD-L1 therapy resistance by limiting innate immunity and tumoral ferroptosis. J Clin Invest (2021) 131(8):e139434. doi: 10.1172/JCI139434 PMC8262501 33855973

[23] Ye Z Zhuo Q Hu Q Xu X Mengqi L Zhang Z. FBW7-NRA41-SCD1 axis synchronously regulates apoptosis and ferroptosis in pancreatic cancer cells. Redox Biol (2021) 38:101807. doi: 10.1016/j.redox.2020.101807 33271455 PMC7710650

[24] Luis G Godfroid A Nishiumi S. Tumor resistance to ferroptosis driven by stearoyl-CoA desaturase-1 (SCD1) in cancer cells and fatty acid binding protein-4 (FABP4) in tumor microenvironment promote tumor recurrence. Redox Biol (2021) 43:102006. doi: 10.1016/j.redox.2021.102006 34030117 PMC8163990

[25] Li M Jin S Zhang Z. Interleukin-6 facilitates tumor progression by inducing ferroptosis resistance in head and neck squamous cell carcinoma. Cancer Lett (2022) 527:28–40. doi: 10.1016/j.canlet.2021.12.011 34902522

[26] Han F Li S Yang Y. Interleukin-6 promotes ferroptosis in bronchial epithelial cells by inducing reactive oxygen species-dependent lipid peroxidation and disrupting iron homeostasis. Bioengineered (2021) 12(1):5279–88. doi: 10.1080/21655979.2021.1964158 PMC8806540 34402724

[27] Zhu G Sui S Shi F. Inhibition of USP14 suppresses ferroptosis and inflammation in LPS-induced goat mammary epithelial cells through ubiquitylating the IL-6 protein. Hereditas (2022) 159(1):21. doi: 10.1186/s41065-022-00235-y 35549778 PMC9102600

[28] Xiao Y Yu D. Tumor microenvironment as a therapeutic target in cancer. Pharmacol Ther (2021) 221:107753. doi: 10.1016/j.pharmthera.2020.107753 33259885 PMC8084948

[29] Zalfa C Paust S. Natural killer cell interactions with myeloid derived suppressor cells in the tumor microenvironment and implications for cancer immunotherapy. Front Immunol (2021) 12:633205. doi: 10.3389/fimmu.2021.633205 34025641 PMC8133367

[30] Takeda A Homma S Okamoto T Kufe D Ohno T. Immature dendritic cell/tumor cell fusions induce potent antitumour immunity. Eur J Clin Invest (2003) 33(10):897–904. doi: 10.1046/j.1365-2362.2003.01194.x 14511362

[31] Melillo RM Guarino V Avilla E Galdiero MR Liotti F Prevete N. Mast cells have a protumorigenic role in human thyroid cancer. Oncogene (2010) 29(47):6203–15. doi: 10.1038/onc.2010.348 20729915

[32] Grisaru-Tal S Itan M Grass DG Torres-Roca J Eschrich SA Gordon Y. Primary tumors from mucosal barrier organs drive unique eosinophil infiltration patterns and clinical associations. Oncoimmunology (2020) 10(1):1859732. doi: 10.1080/2162402X.2020.1859732 33457078 PMC7781846

[33] Han N Zhang Z Liu S Ow A Ruan M Yang W. Increased tumor-infiltrating plasmacytoid dendritic cells predicts poor prognosis in oral squamous cell carcinoma. Arch Oral Biol (2017) 78:129–34. doi: 10.1016/j.archoralbio.2017.02.012 28242507

